# Supplementary material for: Gold Nanoparticles Synthesized with Common Mullein (Verbascum thapsus) and Castor Bean (Ricinus communis) Ethanolic Extracts Displayed Antiproliferative Effects and Induced Caspase 3 Activity in Human HT29 and SW480 Cancer Cells
Source: Pharmaceutics. 2022 Sep 28;14(10):2069. doi: 10.3390/pharmaceutics14102069 (PMC9609588; doi:10.3390/pharmaceutics14102069)
Supplement: Supplementary file 1 [file pharmaceutics-14-02069-s001.zip › pharmaceutics-1928310-supplementary.pdf]

pharmaceutics

Supplementary Materials: Gold Nanoparticles Synthesized with Common Mullein (*Verbascum thapsus*) and Castor Bean (*Ricinus communis*) Ethanolic Extracts Displayed Antiproliferative Effects and induced Caspase 3 Activity in Human HT29 and SW480 Cancer Cells

Karen M. Soto, Ivan Luzardo-Ocampo, José M. López-Romero, Sandra Mendoza, Guadalupe Loarca-Piña and Eric M. Rivera-Muñoz and Alejandro Manzano-Ramírez

Table S1. Individual phenolic compounds' composition by HPLC-DAD or ME and CE extracts.

Compound	Retention time (min)	Concentration (μg equivalents compound/g DW) Common mullein (ME)	Castor bean (CE)
Hydroxybenzoic acids derivatives and benzaldehydes			
Gallic acid	1.80	28.57 ± 0.19 [a]	8.42 ± 0.32 [b]
Hydroxyphenylacetic acid	9.80	15.78 ± 0.11 [a]	16.14 ± 0.07 [a]
Hydroxybenzoic acid	12.33	0.87 ± 0.01 [b]	3.33 ± 0.09 [a]
Hydroxycinnamic acids and derivatives			
Caffeic acid	2.80	3.32 ± 0.04 [b]	37.48 ±0.13 [a]
p-Coumaric acid	4.40	79.93 ± 0.13 [a]	71.88 ±0.11 [b]
Ferulic acid	5.30	19.07 ± 0.08 [b]	310.71 ± 0.39 [a]
Sinapic acid	5.10	12.23 ± 0.06 [b]	19.14 ± 0.91 [a]
Flavonols			
Rutin	3.30	13.31 ± 0.62 [a]	8.14 ± 0.13 [b]
Quercetin	13.30	1.44 ± 0.07 [b]	5.71 ± 0.05 [a]
Flavones			
(+)-Catechin	2.20	20.62 ± 0.23 [a]	12.68 ± 0.35 [b]
Epicatechin	11.55	3.32 ± 0.01 [b]	15.22 ± 0.41 [a]
Epigallocatechin gallate	11.79	5.22 ± 0.91 [b]	87.85 ± 0.17 [a]
Hydrolyzable tannins			
Ellagic acid	3.80	0.60 ± 0.02 [b]	10.59 ± 0.10 [a]

The results are the mean ± SD of three independent experiments in triplicate. Different letters (a, b) by row indicate significant differences ($p < 0.05$) by Tukey-Kramer's test between treatments (ME and CE). CE: Castor bean (Ricinus communis) leaves ethanolic extract; DW: Dry weight; ME: Common mullein (*V. thapsus*) flowers ethanolic extract.

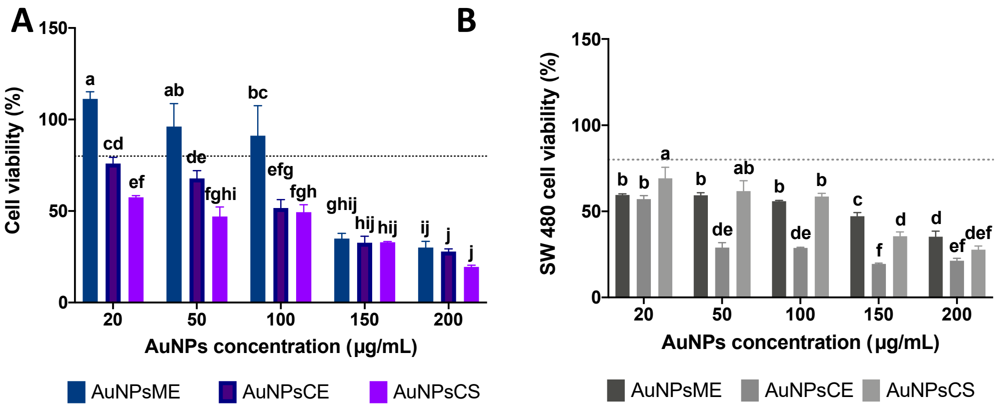

Figure S1. Impact of AuNPs on HT29 (**A**) and SW480 (**B**) cells viability. Different letters in (A) and (B) (a, b, c, d, e, f, g, h, i, and j)express significant differences ($p < 0.05$) by Tukey-Kramer's test. The dashed line indicates 80% viability. AuNPsCE: Gold nanoparticles synthesized with castor bean (Ricinus communis) leaves extract; AuNPsCS: Gold nanoparticles produced by chemical synthesis; AuNPsME: Gold nanoparticles synthesized with common mullein (V. thapsus) flowers ethanolic extract.

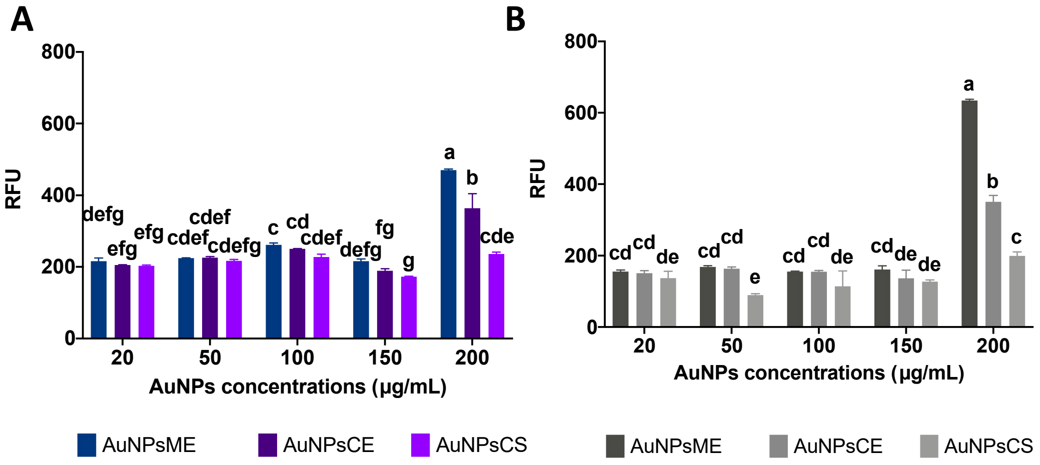

Figure S2. Effect of AuNPs on HT29 (**A**) and SW480 (**B**) ROS generation. Different letters in A and B (a,b,c,d,e,f, and g) express significant differences ($p < 0.05$) by Tukey-Kramer's test. AuNPsCE: Gold nanoparticles synthesized with castor bean (*Ricinus* communis) leaves extract; AuNPsCS: Gold nanoparticles produced by chemical synthesis; AuNPsME: Gold nanoparticles synthesized with common mullein (*V. thapsus*) flowers ethanolic extract**;** RFU: Relative fluorescence units.

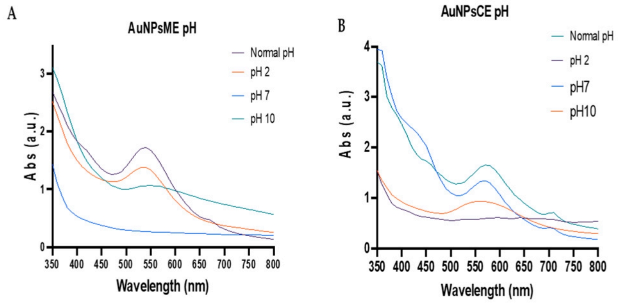

Figure S3. UV-Vis spectra of the effect of pH in the synthesis of AuNPs.
